# Maximal lactate steady state in T53/54 wheelchair racing

**DOI:** 10.7717/peerj.20986

**Published:** 2026-04-07

**Authors:** Lingling Zhang, James R. Broatch, Xueping Wu

**Affiliations:** 1 Physical Education Department, Shanghai University of Finance and Economics, Shanghai, China; 2 Institute for Health & Sport, Victoria University, Melbourne, Victoria, Australia; 3 School of Physical Education and Training, Shanghai University of Sport, Shanghai, China

**Keywords:** Lactate, Anaerobic threshold, Endurance training, Para-athletes

## Abstract

**Background:**

The maximal lactate steady state (MLSS), a physiological state corresponding to the highest workload maintainable over time without continual blood lactate accumulation, is widely used to assess endurance performance and formulate training programs. However, currently accepted values for MLSS are based on non-disabled individuals and may be unsuitable for wheelchair racers. This study measured the blood lactate concentration (BLC) at MLSS and the corresponding load for class T53/54 wheelchair racers. Additionally, the validity of using a fixed BLC for calculating the workload at MLSS was assessed.

**Methods:**

Nine elite T53/54 wheelchair racers (23.7 ± 4.8 years, 59.4 ± 8.6 kg, training time 4.4 ± 2.7 years) were included in a 1,500-m simulation test, a six-level incremental test, and two to five 30-min submaximal constant load tests on an out door standard 400-m track. Earlobe BLC, heart rate, and velocity were measured. MLSS represented the highest BLC with an increase of ≤ 1.0 mM during the final 20 min of the constant load tests.

**Results:**

The BLC at MLSS was 5.3 ± 1.1 mM. The speed measured at MLSS was significantly higher than that measured during the incremental test at BLC = 4 mM (*P* < 0.05), but similar to that at 5.3 mM. The rating of perceived exertion at MLSS was 12.9 ± 1.1, and the velocity at MLSS was 80.6 ± 2.5% of the maximum workload corresponding to the incremental test.

**Conclusions:**

A BLC of 5.3 mM is a more accurate ‘group’ estimate for wheelchair racers than the four mM threshold identified in non-disabled individuals. Furthermore, this intensity corresponds to approximately 80.6% of maximal workload and a rating of perceived exertion (RPE) of 13, providing a multi-faceted, sport-specific method for prescribing training.

## Introduction

The maximal lactate steady state (MLSS) represents the highest blood lactate concentration and work load that can be maintained without continual blood lactate accumulation (Billat et al., 2003; Poole et al., 2021). MLSS has been used as a tool to standardize aerobic training intensity, evaluate the aerobic capacity of individual athletes (Beneke & Von Duvillard, 1996), and determine the validity of other anaerobic threshold testing methods (*e.g.*, ventilatory threshold, lactate minimum test, and critical power; Azevedo et al., 2021; Madrid et al., 2016; Ozkaya et al., 2022; Caen et al., 2024; Poole et al., 2021). Direct assessment of MLSS *via* blood lactate concentration (BLC) requires 2–5 30-min sub-maximal constant workload tests on different days, with at least 24 h between the tests (Heck & Wackerhage, 2024; Poole et al., 2021). This protocol is expensive, time-consuming, and requires a high level of cooperation among athletes, coaches, and researchers.

An important limitation in using MLSS for the prescription of exercise intensity is the heterogeneity reported between individuals. In a landmark study involving 16 non-disabled male participants with varying endurance capacities, the MLSS during sub-maximal running was reported to be four mM (range: 3.05–5.52 mM; Heck et al., 1985). This led to a fixed BLC value of four mM (lactate threshold four mM, abbreviated as LT4) being commonly used to prescribe exercise intensity at MLSS for endurance training in both cyclic land and water sport activities. However, reported MLSS values vary considerably between 1.8 to 7.1 mM depending on the exercise modality (Li, Zhang & Li, 2022; Billat et al., 2003). Therefore, when a running-based MLSS is applied to other exercise modalities (*e.g.*, cycling or swimming), the prescribed intensity is probably underestimated or overestimated, resulting in an endurance training intensity that is too high or too low.

Furthermore, physiological standards derived from non-disabled, lower-body-dominant exercise modalities (*e.g.*, running) may not be transferable to wheelchair racing. This stems from fundamental physiological differences: (1) Wheelchair racing relies on a smaller active upper-body muscle mass, which not only limits maximal oxygen uptake but may also result in a smaller lactate distribution space (Bhambhani, 2002; Price, 2010). (2) Upper-body musculature typically possesses a higher percentage of Type II (fast-twitch) fibers compared to the lower limbs (Bhambhani, 2002; Schneider, Wing & Morris, 2002; Bernardi et al., 2010), leading to greater glycolytic rates and lactate production at a given relative intensity. (3) The characteristics of spinal cord injury (SCI) in T53/54 athletes (ranging from T1 to S4) can be associated with autonomic dysfunction, which impacts cardiovascular responses and blood flow to non-active tissues, including the liver, potentially compromising systemic lactate clearance (Price, 2010; Krassioukov, 2009; Wecht et al., 2020). Given that these factors collectively influence lactate kinetics, the applicability of the traditional four mM threshold to T53/54 athletes is highly questionable.

This study aimed to investigate the BLC and workload at MLSS in T53/54 wheelchair racers. The athletes in these classes have eligible impairments such as limb deficiency, impaired muscle power, and leg discrepancy, with trunk muscle power ranging from absent to normal. These impairments may result from underlying health conditions including amputation, poliomyelitis, or spinal cord injuries (ranging from neurological level T1 to S4; WPA, 2024). Therefore, this study aimed to directly determine the MLSS in elite T53/54 wheelchair racers. The central research question was to investigate whether the MLSS in wheelchair athletes aligns with the four mM threshold commonly applied in non-disabled sports or if wheelchair racing necessitates a distinct, discipline-specific criterion. Answering this research question is crucial to developing evidence-based training guides in Paralympic wheelchair racing.

## Methods

### Participants

Nine T53/54 wheelchair racers (eight male and one female, age 23.7 ± 4.8 years, sitting height 89.0 ± 5.6 cm, weight 59.4 ± 8.6 kg, time post-injury 17.8 ± 8.2 years) chose to be involved in this study. Six of the participants were in the T54 class (amputees, victims of poliomyelitis, and SCI at T8 to S4), and the remaining three were in the T53 class (SCI at T1 to T7). To make the cohort more homogeneous, athletes with T51/52 (high-level paraplegic) or T33/T34 (cerebral palsy) disabilities were excluded from the study (Table 1). At the time of the study, all nine participants had performed 15 to 20 h of systematic training per week for 4.4 ± 2.7 years and had international competition experience. The study procedures were described to the participants and their coaches, and conducted in accordance with the Declaration of Helsinki. All athletes provided written informed consent prior to participating in the experiment. The study received ethical approval from the Academic Committee of Shanghai University of Sport (102772024RT081).

**Table 1:** Physical characteristics of the participants.

**No.**	**Class**	**Sex**	**Injury**	**Age**	**Sitting height**	**Weight**	**Arm length**	**Time post-injury**	**Training time**
				**(y)**	**(cm)**	**(kg)**	**(cm)**	**(y)**	**(y)**
1	T54	F	Amp.^a^	31.0	78.0	78.0	170.0	30.0	10.0
2	T54	M	Polio^b^	18.0	95.0	54.0	180.0	17.0	2.0
3	T54	M	Amp.^a^	22.0	90.0	56.0	170.0	4.0	4.0
4	T54	M	Amp.^a^	24.0	94.0	61.0	184.0	20.0	7.0
5	T54	M	Polio^b^	20.0	90.0	65.7	176.5	20.0	3.0
6	T54	M	SCI^c^	24.0	89.0	53.0	185.0	24.0	2.0
7	T53	M	SCI^c^	22.0	90.0	49.5	166.0	6.0	3.0
8	T53	M	SCI^c^	32.0	93.0	62.0	172.0	21.0	6.0
9	T53	M	Polio^b^	20.0	82.0	55.0	166.0	18.0	3.0
Mean	–	–	–	23.7	89.0	59.4	174.4	17.8	4.4
SD^d^	–	–	–	4.8	5.6	8.6	7.3	8.2	2.7

**Notes.**

a Amp., amputee.

b Polio, poliomyelitis.

c SCI, spinal cord injury.

d SD, standard deviation.

### Test procedure

The participants used their own racing wheelchairs for a 1,500-m simulated race test, an incremental exercise test (IET), and 2–5 30-min constant load tests (CLTs). All tests were performed on separate days (>24 h apart) on a standard outdoor 400-m athletic track. Participants were advised to avoid strenuous physical exercise during the two days before the first test to prevent glycogen depletion. Participants were instructed to maintain their diet during each testing day.

All tests were performed between 8:00 and 12:00 or between 13:30 and 18:00. To reduce diurnal variation, all intra-individual tests were performed at the same time of the day. The researchers were proficient with the testing procedures, all instruments were adequately calibrated, and the same blood lactate analyzer and blood collector were used for each participant to reduce possible errors in sample collection and analysis (Faude, Kindermann & Meyer, 2009). The average temperature, humidity, and atmospheric pressure during the tests were 22.2 ± 2.8 °C, 72.4 ± 11.4%, and 1,025.5 ± 12.59 hPa, respectively.

### Instrumentation

To determine the BLC, 10-µl blood samples were collected from the earlobe using inhibitor tubes pre- and post-warm-up, after each level, and at 1, 3, 5, 7, and 10 min following the IET and CLTs. All samples were immediately placed on ice and batch-analyzed within two hours using a lactate analyzer (Biosen C-Line; EKF Diagnostic, Barleben, Germany) that was calibrated daily per manufacturer guidelines, following validated protocols for pre-analytical stability (Seymour et al., 2011; Zavorsky et al., 2020). The Biosen C-Line analyzer used has a reported intra-assay CV of < 2.0% for lactate measurements (Seymour et al., 2011; Zavorsky et al., 2020) During the 1,500-m simulated race test, IET, and CLTs, participants wore a heart rate band (Polar Accurex Plus; Polar Electro Oy, Kempele, Finland) and a GPS tracking device (GPSports) to continuously monitor heart rate (HR) and speed during all tests. The rating of perceived exertion (RPE) was directly evaluated after each stage of the IET and CLT using the Borg-scale 6–20 (Borg, 1982).

### Exercise testing

#### 1,500-m simulation test

The 1,500-m race distance was selected as the reference benchmark for several key reasons. First, it is a standardized official event for T53/T54 wheelchair athletes. Second, it represents a highly relevant, aerobically dominant intensity for this population; the typical completion time of the 1,500-m race (≈ 2:55–3:10 in elite T53/54 athletes) places it in a high-intensity aerobic domain, and prior research confirms that the majority of the athlete’s energy during this event (*e.g.*, 71.7 ± 7.1%) is derived from aerobic metabolism (Zhang, Wu & Li, 2018). Finally, using the 1,500-m Vmean as the 100% baseline follows the methodology established by Zhang, Wu & Li (2018), allowing the IET stages to be normalized to each athlete’s maximal performance. This concept is further supported by studies (*e.g.*, De Lucas et al., 2012) demonstrating the utility of 1500-m performance in informing aerobic thresholds.

All athletes completed the 1,500-m test on an outdoor track using their own wheelchair. Each athlete’s average speed (V_mean_) during this test was used as the intensity criterion for the individual IET.

### Incremental exercise test (IET)

The IET comprised of five 5-min stages, with 1-min intervals between stages for blood sampling. The participants and their RPE, heart rate (HR), and speed were monitored during the entire test. After a 15-min self-selected and self-paced warm up at low intensity, the participants were instructed to sit quietly in their wheelchairs for 5 min while the HR and GPS equipment was set up. The five stages of the IET were performed at 75%, 80%, 85%, 90%, and 95% of the V_mean_. Audio prompts were provided to the participants 30 s before starting each stage, at the beginning of each stage, at every 50-m interval, and at the end of each stage. In addition, the 400-m track was set up with markers at every 50 m, and the racing wheelchairs were equipped with real-time speed displays. The participants were asked to comply with the audio cues and the established speed requirements as much as possible during the test.

### Constant load tests (CLTs)

**Figure 1:**
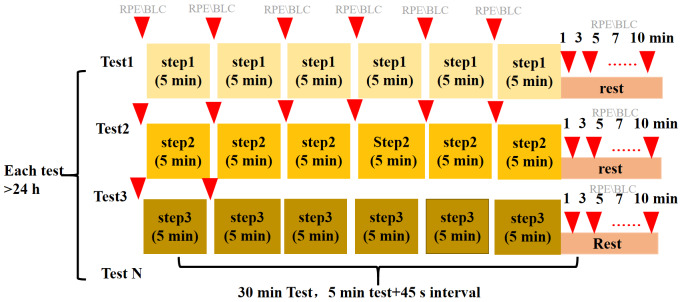
Procedure of the sub-maximal constant load test. Each participate constant load test (CLT) comprised 6 × 5 min stages, with a 45 s break between the stages for blood lactate sampling and assessment of RPE. RPE, rating of perceived exertion on Borg’s 6–20 RPE scale (Borg, 1982); BLC: blood lactate concentration.

All CLTs lasted 30 min unless terminated owing to volitional fatigue. Each CLT comprised of six 5-min stages, with a 45 s break between the stages for blood sampling and assessment of RPE (Fig. 1). The speed of the first CLT was based on the velocity corresponding to BLC = 4 mM calculated from the IET (V-LT4) *via* linear interpolation. The velocity in the following CLTs was −1.5 to +5% higher or lower. For example, if a participant’s speed during the first 30-min CLT was 25 km/h, then the speed range for the subsequent 30-min CLT would be 24.625–26.25 km/h depending on the BLC in the previous CLT. Following the criteria of Beneke (2003), MLSS was determined as the maximal workload where the BLC increase remained within ± 1.0 mM during the last 20 min of the CLTs. The corresponding velocity was defined as the MLSS workload (MLSS-V; Beneke et al., 1996).

**Figure 2:**
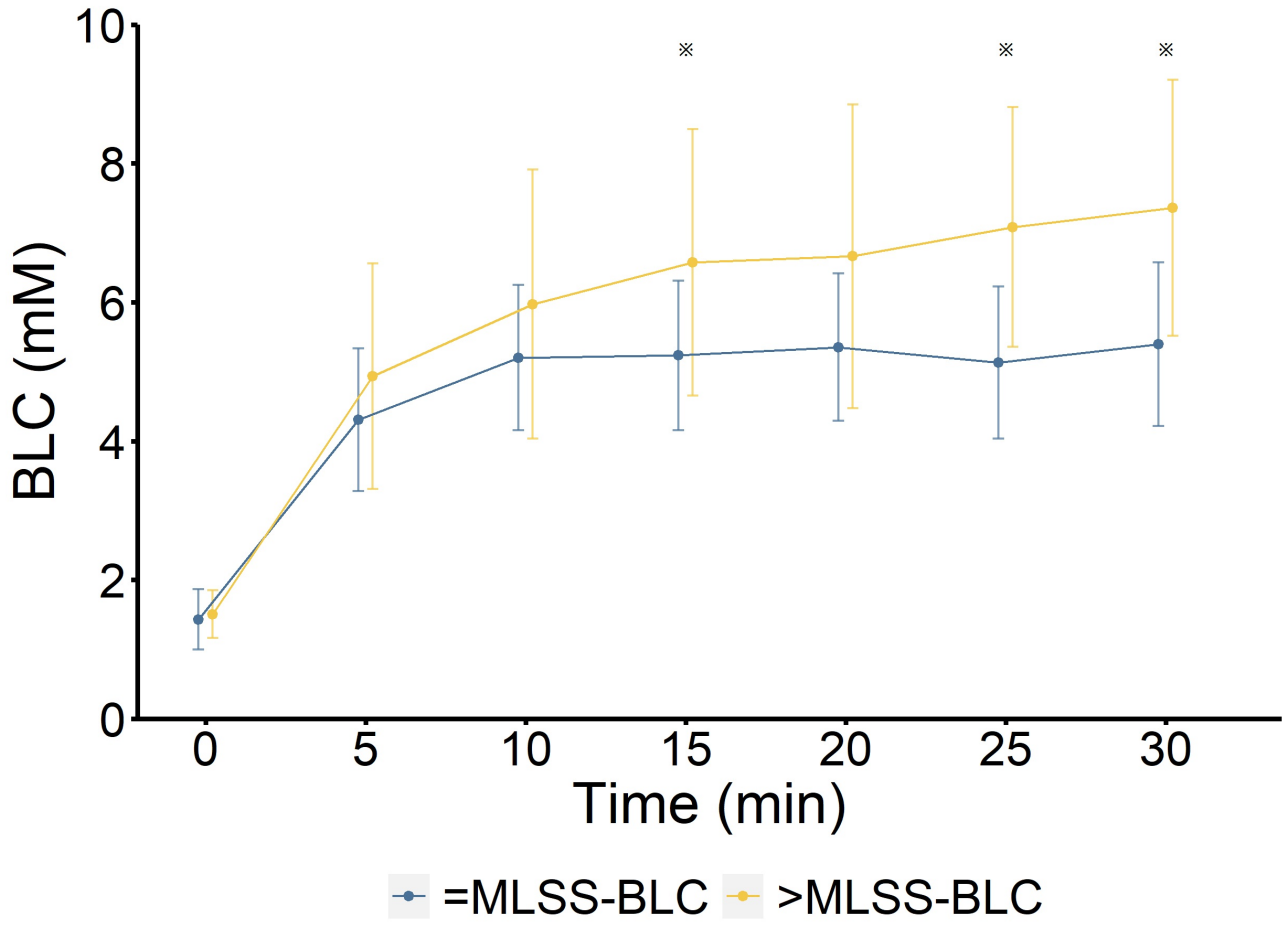
Blood lactate concentration (BLC) at MLSS workload and above MLSS workload. MLSS-BLC (blue): BLC at MLSS workload; >MLSS-BLC (yellow): BLC at above MLSS workload (*n* = 9). ※  indicates a significant difference between MLSS-BLC and >MLSS-BLC (*P* < 0.05). The velocity at MLSS workload is 23.9 ± 1.3 km/h, and that above MLSS workload is 24.5 ± 1.4 km/h.

### Statistical analysis

Data analysis was carried out using SPSS Statistics 26 (IMB Corporation, USA). To analyze the blood lactate concentration (BLC) changes during the 30-min constant load tests (CLTs; Fig. 2), the two-way repeated measures analysis of variance (ANOVA) was conducted with factors of condition (MLSS *vs.* > MLSS) and time (seven time points: 0, 5, 10, 15, 20, 25, 30 min). Mauchly’s test of sphericity was applied. As sphericity was violated for the main effect of time (*P* < 0.001) and the Condition × Time interaction (*P* = 0.027), the Greenhouse-Geisser correction was used. Significant interaction effects were explored using simple effects analysis with adjustments for multiple comparisons.

The interpolation method (Heck et al., 1985; Mader et al., 1976) was used to calculate the speed corresponding to an individual’s BLC value of four mM (the value fixed by Heck) and 5.3 mM (MLSS-BLC determined in the present study for wheelchair racing) during IET. The corresponding equation is:

\begin{eqnarray*}y= \frac{(y2-y1)\ast (x-x1)}{(x2-x1)} +y1. \end{eqnarray*}

For example, when the BLC value of 4 mM appeared between the 2nd and 3rd stages in the IET, then *x* = 4, *x*1 is the BLC of the 2nd stage, *x*2 is the BLC of the 3rd stage, *y*1 is the average speed of the 2nd stage, *y*2 is the average speed of the 3rd stage, and *y* is the interpolated speed when the individual should reach BLC = 4 mM in the IET. The interpolation for BLC = 5.3 mM was performed in the same way. These interpolated speeds (V-LT4 and V-LT5.3) and the directly measured MLSS-V were compared using a one-way repeated measures ANOVA, with measurement type (MLSS-V, V-LT4, V-LT5.3) as the within-subjects factor. A Tukey’s honestly significant difference (HSD) *post-hoc* test was used to identify specific differences between the speed measurements. A significance level was set at *α* = 0.05.

A Bland–Altman analysis and the concordance correlation coefficient (CCC) were used to assess the agreement between the velocities derived from fixed blood lactate thresholds *via* IET interpolation (V-LT4 and V-LT5.3) and the gold-standard MLSS-V. The Bland–Altman analysis was used to calculate the mean bias and the 95% limits of agreement (LOA) between the two methods. The CCC was used to evaluate the degree of concordance between the two measurement methods. All data are presented as mean and standard deviation.

## Results

The IET was completed on an outdoor track in five stages. The intensity achieved at each stage was 76.4 ± 1.3%, 81.0  ± 1.6%, 85.3 ± 1.1%, 89.6 ± 1.6%, and 95.3 ± 3.6% of the average speed of the 1500-m test, respectively. At stages 1–5, the average speed was 20.3 ± 2.2, 21.6 ± 2.3, 22.9 ± 2.4, 24.2 ± 2.6, and 25.5 ± 2.7 km/h, respectively; and the corresponding BLC was 2.4 ± 1.1, 2.7 ± 1.4, 4.0 ± 2.1, 6.1 ± 3.3, and 8.7 ± 3.9 mM, respectively.

Eight participants completed the IET, and their speed and BLC at each stage are listed in Table 2. The BLC value of 4 mM was observed at stages 2 to 3, meeting the test requirements. All nine participants completed a 30-min CLT exceeding the MLSS load (Table 3).

**Table 2:** Five stage incremental exercise test velocity and BLC indicator.

	Unit	1st stage	2nd stage	3rd stage	4th stage	5th stage
Speed^a^	km/h	20.3 ± 2.2	21.6 ± 2.3	22.9 ± 2.4	24.2 ± 2.6	25.5 ± 2.7
1,500 m V_mean_^b^	%	76.4 ± 1.3	81.0 ± 1.6	85.3 ± 1.1	89.6 ± 1.6	95.3 ± 3.6
BLC^c^	mM	2.4 ± 1.1	2.7 ± 1.4	4.0 ± 2.1	6.1 ± 3.3	8.7 ± 3.9

**Notes.**

a The speed for each incremental exercise test.

b The intensity for each incremental exercise test is a percentage of the average speed from the participants full-effort 1,500 m test.

c The blood lactate value corresponding to the 5 stages incremental exercise test.

**Table 3:** MLSS results for the participants.

Item		Unit	Mean	SD	Max	Min
MLSS-BLC^a^		(mM)	5.3	1.1	6.5	3.6
MLSS-V_mean_^b^		(km/h)	23.9	1.3	25.6	22.0
MLSS-V_peak_^c^		(km/h)	26.4	2.0	30.0	24.0
MLSS-HR_ave_^d^		(bpm)	159.0	10.3	180.0	147.8
MLSS-HR_peak_		(bpm)	169.3	11.1	191.0	155.5
V-LT4^e^		(km/h)	22.6	1.8	24.2	18.9
V-LT5.3		(km/h)	23.9	1.9	25.6	19.3
V_max_^f^		(km/h)	28.9	1.9	31.0	25.2
%V_max_^g^		(%)	80.6	2.5	85.5	78.2
RPE		(6–20)	12.9	1.1	15.0	12.0

**Notes.**

a MLSS-BLC: BLC at MLSS

b MLSS-V_mean_: average velocity under MLSS intensity.

c MLSS-V_peak_: peak velocity under MLSS intensity.

d MLSS-HR_ave_: average heart rate under MLSS intensity.

e V-LT4: velocity corresponding to 4-mM BLC calculated for an individual in incremental exercise test.

f V_max_: fastest speed in the incremental exercise test.

g %V_max_: highest intensity percentage of increasing load corresponding to MLSS intensity.

MLSS-BLC for the T53/54 wheelchair racers was determined to be 5.3 ± 1.1 mM (range: 3.6–6.5 mM), and the corresponding MLSS-V reached 80.6 ± 2.5% of the maximum workload in the IET (range: 78.2–85.5%). The MLSS-HR_ave_ was 159.0 ± 10.3 bpm (range: 147.8–180.0 bpm), and the MLSS-HR_peak_ was 169.3 ± 10.1 (range: 155.5–191.0 bpm). The RPE was 12.6 ± 0.7 (range: 12–14).

Analysis of the 30-min CLT (Fig. 2) using a two-way repeated measures ANOVA revealed a significant main effect for condition *F* (1, 8) = 8.05, *P* = 0.022, *η*^2^ = 0.154) and a significant main effect for time (Greenhouse-Geisser corrected: *F* (1.73, 13.87) = 75.67, *P* < 0.001, *η*^2^ = 0.583). The Condition × Time interaction was significant (*F* (2.77,22.13) = 4.67, *P* < 0.05, *η*^2^ = 0.055). A simple effects analysis to further assess this interaction showed no significant difference in BLC between the MLSS and > MLSS conditions at baseline or at 5, 10, 15, and 20 min (all *P* > 0.03). However, BLC was significantly higher in the > MLSS condition at 25 min (*P*_adj_ = 0.007) and 30 min (*P*_adj_ = 0.007).

**Figure 3:**
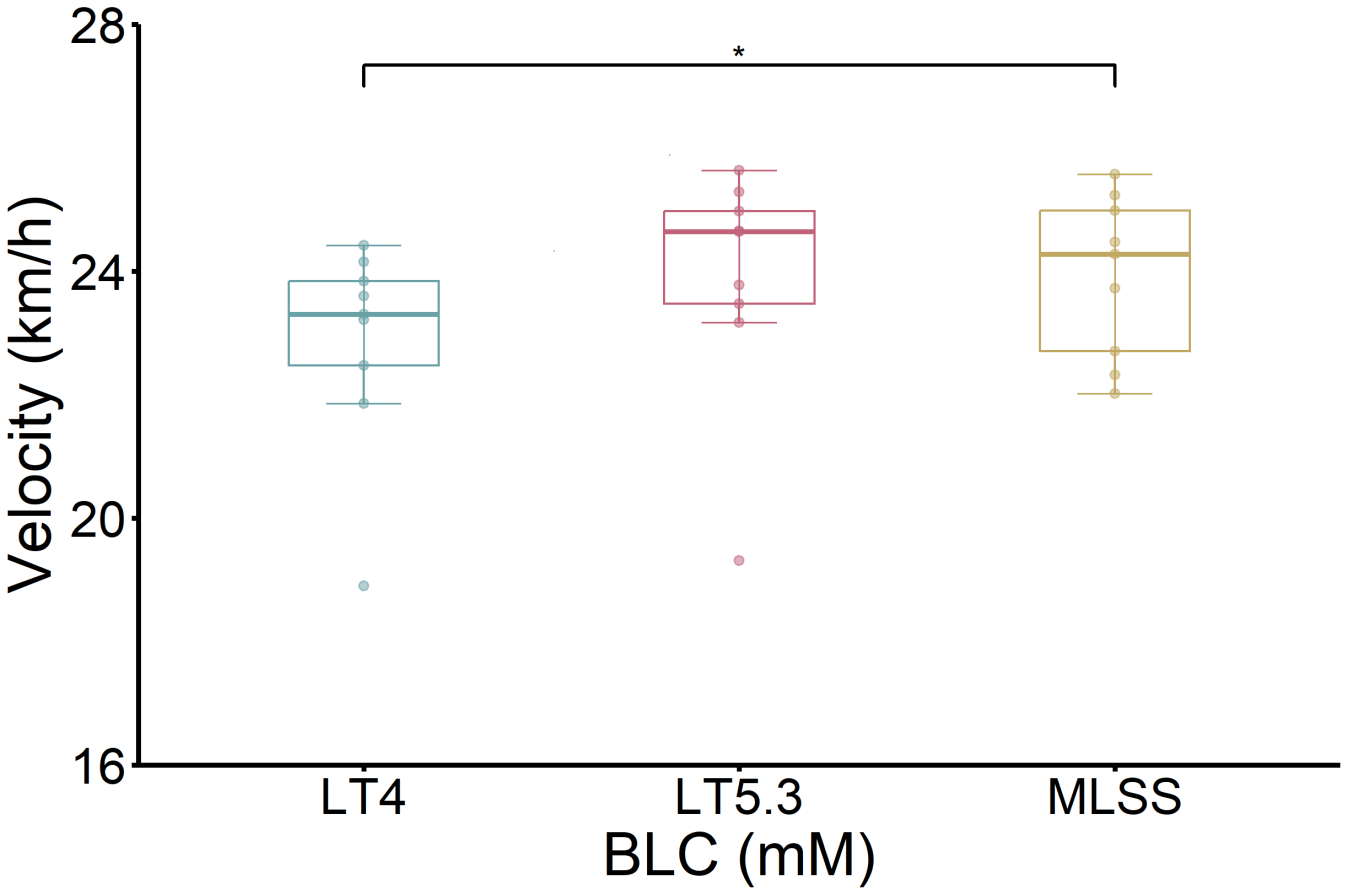
Comparison of MLSS between V-LT4 and V-LT5.3. V-LT4 and V-LT5.3 (first and second bars): velocities corresponding to BLC = 4 and 5.3 mM, respectively. Data for each participant was calculated by interpolation from the 5-stage IET. An asterisk (*) indicates a significant difference between V-LT4 and V-LT5.3 (*P* < 0.05).

There was also a significant difference between MLSS-V and the velocity at LT4 (V-LT4, Fig. 3), namely 23.9 ± 1.3 *vs.* 22.9 ± 1.7 km/h (*P* < 0.05). Importantly, V-LT4 was significantly different from V-LT5.3 (23.9 ± 1.9 km/h, *P* < 0.05). In addition, no statistically significant association was observed between MLSS-BLC and LT5.3 (*P* > 0.05).

To further evaluate the validity of V-LT4 and V-LT5.3 as surrogates for MLSS-V, an agreement analysis was conducted. The Bland–Altman analysis (Figure Bland–Altman.1) revealed a mean bias of 1.060 km/h between MLSS-V and V-LT4, with 95% limits of agreement (LOA) of (−1.670, 3.790) km/h. This confirms that V-LT4 systematically underestimates MLSS-V. In contrast, the mean bias between MLSS-V and V-LT5.3 was near zero (0.043 km/h), but the 95% LOA were wide (Figure Bland–Altman.2) at (−2.851, 2.938) km/h, indicating large random individual error.

The CCC analysis confirmed these findings. Agreement between MLSS-V and V-LT4 was poor (CCC = 0.45, 95% CI [−0.08–0.78]). Agreement between MLSS-V and V-LT5.3 was similarly poor (CCC = 0.59, 95% CI [0.01–0.87]), indicating that neither method serves as a reliable surrogate for MLSS-V.

The one-way repeated measures ANOVA revealed a significant main effect for measurement type (*F* (2, 16) = 4.53, *P* = 0.028). *Post-hoc* analysis using the Tukey’s HSD test indicated that a statistical difference existed between MLSS-V and V-LT4 (*P* = 0.043).

## Discussion

Previous studies have reported that MLSS varies from one sport to another depending on the motor pattern. This study aimed to determine whether the fixed LT4 could be used as an anaerobic threshold for wheelchair racing training by directly measuring the MLSS of elite wheelchair racers.

### Characteristics of MLSS-BLC for wheelchair racing

The principal finding of this study is that the MLSS for elite T53/54 wheelchair racers occurs at a BLC of 5.3 ± 1.1 mM. This value is substantially higher than the widely cited 4 mM fixed lactate threshold established in many non-disabled sports (Heck et al., 1985; Mader et al., 1976). Despite the heterogeneity in age, gender, and training experience among our participants, the primary determinant of the group mean MLSS-BLC (5.3 mM) appears to be the sport-specific, upper-body-dominated locomotion pattern (Billat et al., 2003; Hoffman et al., 1996). This finding is supported by literature indicating that MLSS-BLC is independent of gender (Beneke et al., 2009), age within adulthood (Beneke et al., 2009; Beneke et al., 1996), and athletic performance level (Beneke, Hütler & Leithäuser, 2000). The robustness of this central finding—a markedly higher group mean MLSS-BLC in wheelchair racing compared to the 4 mM threshold derived from running—strongly suggests that the physiological demands of upper-body-dominated locomotion exert a far greater influence on lactate kinetics than the inter-individual variability introduced by the composition of this study’s cohort.

The elevated MLSS-BLC (5.3 mM) observed in this study, compared to the traditional four mM value, can be attributed to the fundamental physiological differences between upper-body dominant exercise and traditional lower-body dominant exercise. This finding is consistent with the consequences of: (1) a smaller active muscle mass leading to a reduced lactate distribution space (Hoffman et al., 1996; Bhambhani, 2002; Price, 2010); (2) a higher proportion of glycolytic type II fibers in the upper-body musculature (Johnson et al., 1973; Beneke & Von Duvillard, 1996; Gladden, 2000), potentially amplified by SCI-induced “slow-to-fast” fiber transitions, leading to greater lactate production (Bhambhani, 2002; Schneider, Wing & Morris, 2002; Xu et al., 2023) and a longer fast component of oxygen uptake kinetics (Pendergast, 1989; Koppo, Bouckaert & Jones, 2002), and (3) a compromised lactate clearance capacity, resulting from both sympathetically-driven splanchnic vasoconstriction during arm exercise (Van Hall et al., 2003) and underlying autonomic dysfunction in SCI athletes (Krassioukov, 2009; Wecht et al., 2020). This combination of higher production, smaller distribution, and reduced clearance provides a robust physiological interpretation for why the lactate steady state occurs at a higher concentration in this population.

In summary, a higher rate of lactate production (from fast-twitch fibers), combined with a smaller distribution space (from smaller muscle mass) and a compromised clearance capacity (due to reduced liver blood flow and autonomic dysfunction), provides a robust physiological rationale for why the balance between lactate appearance and disappearance (MLSS) occurs at a significantly higher concentration in wheelchair racers. This explains why the MLSS-BLC observed in this study (5.3 mM) is higher than that reported for non-disabled running (4.02 mM) (Heck et al., 1985), rowing (2.95–3.1 mM) (Beneke et al., 1996; Beneke, 1995) and swimming (3.5–4.8 mM) (Espada et al., 2021; Dekerle et al., 2005), but aligns closely with other upper-body dominant sports, such as kayaking (5.4 ± 0.7 mM; Li et al., 2014) and para-handcycling (5.1 ± 1.6 mM; Stangier et al., 2019).

We are aware of only one other study on MLSS in wheelchair racing (Perret et al., 2012), in which eight well-trained wheelchair athletes were monitored during an HR-based lactate minimum test (LMHR). The results showed that the HR at lactate minimum (the workload level at which lactate accumulation and removal reach steady-state) was eight to nine bpm lower than the HR at MLSS. In addition, their reported MLSS-BLC of 4.1 ± 2.2 mM was lower than the value determined in the present study, potentially due to variations in locomotor mode, the method of calculation, the testing environment (laboratory treadmill *vs.* outdoor field)—as laboratory based tests may not always elicit maximal physiological responses in highly-trained para-athletes (West et al., 2016)—instrumentation used, and test protocol (non-constant load test based on HR *vs.* constant load test based on lactate). In a study of non-disabled runners and cyclers, the observed HR, power output, oxygen uptake, BLC, and RPE at LMHR were significantly lower than those reported for MLSS (*P* < 0.05; Perret & Hartmann, 2021). As such, the LMHR may not accurately reflect the MLSS intensity, and the issue could be further complicated by the lack of standardization in testing methodology (Carter, Jones & Doust, 1999).

### Characteristics of MLSS intensity for wheelchair racing

MLSS intensity is typically expressed as a percentage of the maximal workload anchor (Beneke, Leithäuser & Hütler, 2001), which is often the peak velocity or power. In this study, the MLSS-V of wheelchair racers was 23.9 ± 1.3 km/h, which corresponded to 80.6 ± 2.5% of the maximum workload achieved during the IET. A one-way repeated measures ANOVA was conducted to compare the velocities derived from the three methods, revealing a significant main effect for measurement type (F (2, 16) = 4.53, *P* = 0.028). *Post-hoc* analysis confirmed that MLSS-V was significantly higher than V-LT4 (*P* = 0.043), establishing that the traditional 4 mM threshold systematically underestimates the true maximal lactate steady state intensity in this population. In contrast, no significant difference was found between MLSS-V and V-LT5.3 (*P* = 0.994). While the comparison between V-LT5.3 and V-LT4 did not reach statistical significance (*P* = 0.053), the trend suggests a meaningful physiological distinction that may achieve significance in a larger cohort.

This finding was further elucidated by an agreement analysis. The Bland–Altman analysis revealed a mean bias of 1.060 km/h between MLSS-V and V-LT4, with 95% limits of agreement (LOA) of (−1.670, 3.790) km/h, quantifying the systematic underestimation by the fixed 4 mM threshold. In contrast, the mean bias between MLSS-V and V-LT5.3 was negligible (0.043 km/h), indicating that V-LT5.3 is accuracy at the group level. However, the 95% LOA for this comparison were wide (−2.851, 2.938) km/h, highlighting substantial random error at the individual level. The CCC analysis corroborated these findings, showing poor agreement for both V-LT4 (CCC = 0.45) and V-LT5.3 (CCC = 0.59) against the gold-standard MLSS-V.

The practical implication of this finding is critical. While V-LT5.3 is an accurate estimator of average MLSS-V for the group, the range of individual error is too large for precise, individual training prescription. Therefore, we conclude that LT5.3 is far superior to LT4 as a ’group’ average estimate, but LT5.3’s high individual variability prevents it from reliably replacing a directly determined MLSS-V for crafting programs for individual athletes. The establishment of an accurate MLSS intensity is crucial, as one study showed that the MLSS intensity is a reliable indicator of endurance capacity across the sub-threshold to supra-threshold range. Consequently, MLSS intensity can be used to adjust training intensity in moderate to severe exercise zones (Hering & Stepan, 2021). Therefore, the methodology for determining this threshold is of practical importance.

This study used an incremental testing protocol consisting of 5-min stages with 1-min intervals. Previous studies found that the lactate threshold itself is affected by the incremental loading test method (*i.e.,* duration of each stage and interval; (Heck et al., 1985). For instance, two studies on running found that the LT4 workload decreases with increasing test duration at each stage (3, 5, and 7 min) and with shorter intervals between the stages (1.5, 1.0, and 0.5 min; Heck et al., 1985; Mader et al., 1976). Rowing tests also showed that incremental intensity at the 4 mM BLC threshold for 3 min per stage can result in a bias towards higher intensities; to circumvent this, the test duration should be adjusted to 8 min (Li et al., 2014). Therefore, coaches and researchers should consider a multi-stage incremental loading test protocol when using LT5.3 workload as the MLSS intensity for T53/54 wheelchair racing.

In this study, the CLT protocol incorporated 45-s pauses every 5 min for blood sampling, which is longer than the 30-s intervals typically used in laboratory settings (Beneke & Von Duvillard, 1996; Grossl et al., 2012). While such interruptions could theoretically permit micro-recovery, the duration of the rest interval during the constant load test can influence recuperation. Related research indicates that the workload of intermittent MLSS is approximately 3∼4% higher than the continuous MLSS in swimming (Dekerle et al., 2009; Greco et al., 2010), 8∼10% higher in cycling (Beneke et al., 2003), and around 6% higher in running (De Lucas et al., 2012). This phenomenon may be attributed to two primary mechanisms: the partial recovery of muscle phosphagens and O_2_ stores, and increased BLC removal during the recovery period (Beneke et al., 2003; Dupont et al., 2004). However, evidence indicates that these interruptions do not significantly alter the determined MLSS-BLC (Beneke, 2003). Therefore, our core finding of an MLSS-BLC at 5.3 mM remains robust. Thus, by better simulating the actual propulsion technique, field testing not only provides a physiologically valid cardiorespiratory and metabolic profile of the athletes (West et al., 2016) but also closely mirrors the demands of competition. As emphasized by Bernardi et al. (2010), this sport-specific and realistic context is the key strength of field assessment, offering superior relevance for devising effective training prescriptions.

### Characteristics of RPE at MLSS-V for wheelchair racing

Because the procedure to measure BLC is costly, complex, and invasive, coaches and athletes may prefer simpler and non-invasive methods to assess MLSS in everyday training settings. For example, the RPE corresponding to the MLSS intensity (4.8 ± 1.5 mM) in swimmers was previously reported to be 13.5 ± 1.5. A linear correlation has also been reported between RPE and intensity indicators for both non-disabled individuals and those with SCI, with no significant differences between these two groups (Goosey-Tolfrey et al., 2014). Our findings demonstrated similar RPE at the MLSS intensity (12.6 ± 0.7) among elite T53/54 wheelchair racers. As such, RPE-13 may be used as a non-invasive proxy to determine MLSS in wheelchair athletes. If a direct MLSS test is not feasible, LT5.3 serves as a better ’starting estimate’ than LT4, but coaches must still fine-tune individual intensity using other metrics, such as RPE (*e.g.*, RPE-13). However, further work is needed to validate this assertion.

This study has several limitations. First, the small sample size (*n* = 9), with only one female participant, reflects the practical challenges in recruiting elite wheelchair racers. This precluded an a priori power analysis, and the findings should be considered preliminary. Future studies should validate these findings using larger and more homogeneous cohorts to enhance the generalizability and robustness of the conclusions. Second, heterogeneity in individual participant characteristics (*e.g.*, sex, age, training history) may have contributed to variability in individual MLSS-BLC values. Third, while our field-based testing on an outdoor standard 400-m track offered high ecological validity (Bernardi et al., 2010), it introduced constraints: (1) speed control was less precise than in laboratory settings, and (2) although ambient temperature and pressure were recorded, other environmental factors such as wind speed were not controlled. Additionally, the constant-load tests employed 45-s stage intervals for blood sampling, which are longer than the 30-s intervals typically used in laboratory settings. Existing evidence suggests that the duration of test intervals may influence the determined MLSS intensity. Future studies should systematically compare different interval modalities (including non-interrupted protocols) to further clarify the optimal testing approach. Finally, the conclusions of this study are primarily derived from a cohort of elite T53/54 athletes; thus, the representativeness and external validity of these findings require further validation with larger sample sizes and studies involving athletes from other classification levels.

## Conclusions

Based on preliminary results, the BLC of 5.3 mM, rather than the traditional 4 mM threshold, provides a more accurate sport-specific estimate of MLSS for T53/T54 wheelchair racers. The findings of this study confirm that LT4 significantly underestimates the true MLSS-V. While LT5.3 serves as a more accurate ‘group’ estimate, the data from this study also highlight the value of non-invasive markers. We conclude that MLSS intensity can be practically estimated using a multi-faceted approach, targeting a BLC of approximately 5.3 mM, which corresponds to roughly 80.6% of maximal workload and an RPE of 13. This combined approach provides a more robust guide for training prescription than using a fixed lactate threshold alone.

### Practical implications

The study provides valuable, practical information for T53/T54 wheelchair racing. Our results verify that the traditionally accepted 4 mM threshold is inappropriate. We recommend coaches and athletes use a multi-faceted approach to prescribe aerobic training. Based on our findings, MLSS intensity corresponds to a BLC of approximately 5.3 mM, which can be non-invasively estimated by targeting approximately 80.6% of the maximal workload (from IET) and/or a rating of perceived exertion (RPE) of 13. Using these three markers (LT5.3, ∼80% max, and RPE 13) in combination provides a practical and more reliable method for guiding training prescription than relying on the 4 mM threshold alone.

## Supplemental Information

10.7717/peerj.20986/supp-1 Supplemental Information 1 IET & CLTs Data The testing environment raw data, the participants blood lactate, velocity, and heart rate, RPE data collected during incremental exercise testing and constant load test.

10.7717/peerj.20986/supp-2 Supplemental Information 2 Supplemental Figures 1 and 2

## Additional Information and Declarations

### Data Availability

The following information was supplied regarding data availability: The raw measurements are available in the Supplemental File.
