# Supplementary figures and images for: Maximal lactate steady state in T53/54 wheelchair racing
Source: PeerJ. 2026 Apr 7;14:e20986. doi: 10.7717/peerj.20986 (PMC13068009; doi:10.7717/peerj.20986)

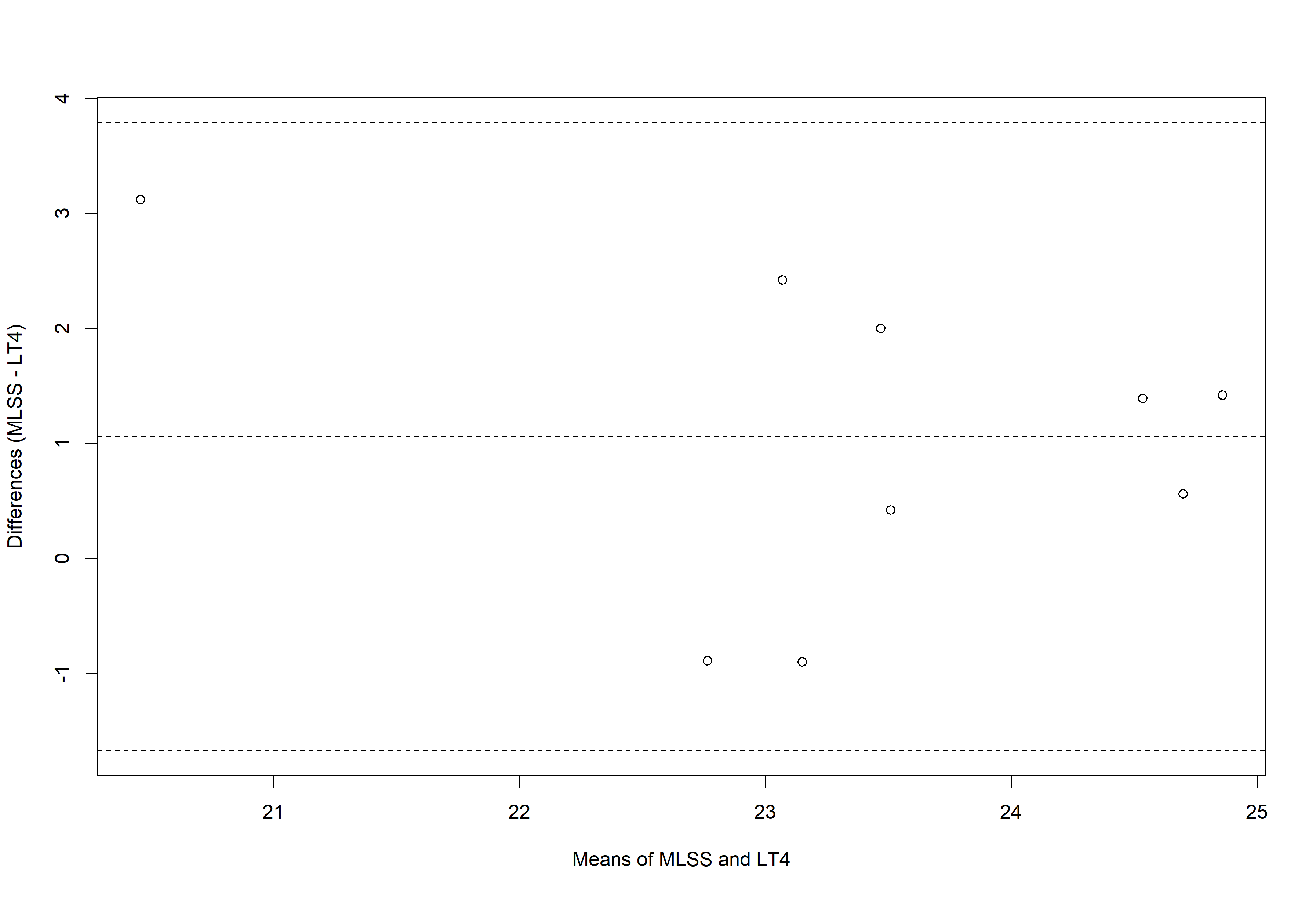


Supplementary Figure Bland-Altman.1


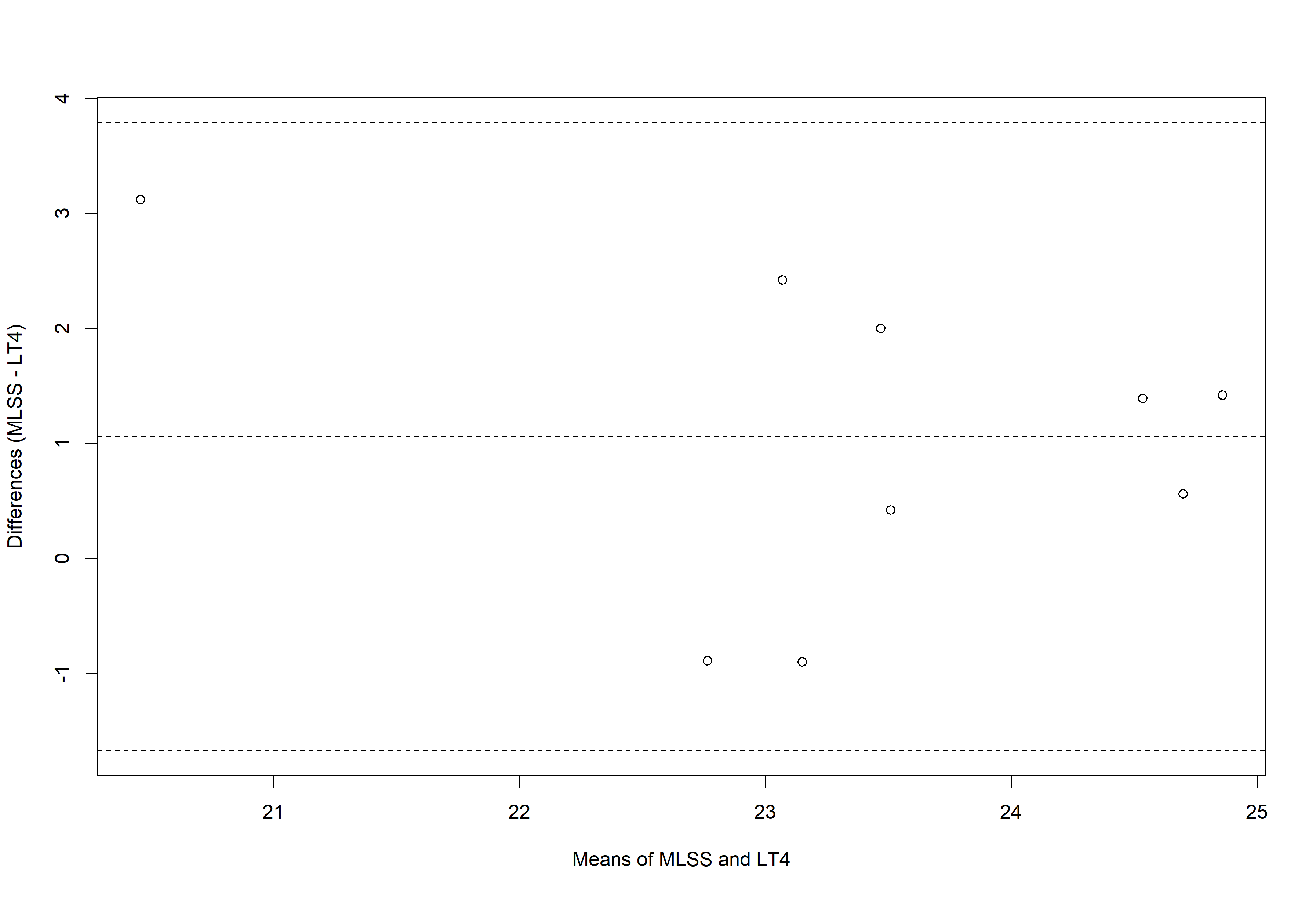


Supplementary Figure Bland-Altman.2

Supplement: Supplemental Information 2 [file peerj-14-20986-s002.docx]
